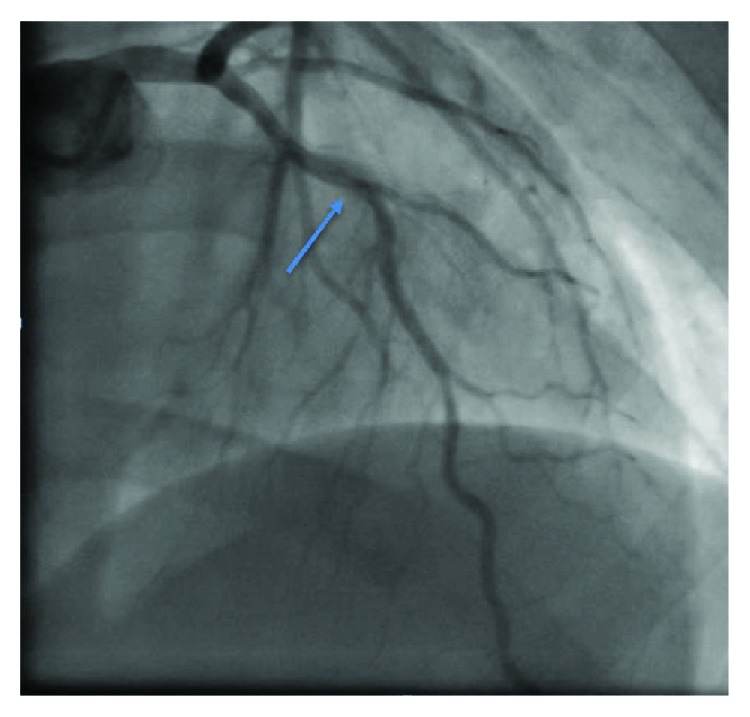# STEMI after Dobutamine Stress Echocardiography in Hyperthyroid State

**DOI:** 10.1155/2019/7434071

**Published:** 2019-04-02

**Authors:** Mahmood Mubasher, Ashfaq Patel, Mohamed Magdi, Tahir Hamid

**Affiliations:** Hamad Medical Corporation, Qatar

## Abstract

Uncontrolled hyperthyroidism has been associated with significant changes in cardiovascular hemodynamics. We report a case of a 39-year-old male who has been recently diagnosed with severe hyperthyroidism. He was undergoing dobutamine stress echocardiography (DSE) for evaluation of symptoms suggestive of stable angina. The exam was complicated by ST-segment elevation myocardial infarction- (STEMI-) required coronary angiography that showed mild coronary artery disease.

## 1. Introduction

It is known that hyperthyroidism alters the hemodynamics of the cardiovascular system through increasing the oxygen demand [1]. Dobutamine stress echocardiography (DSE) is an effective and safe test to detect coronary artery disease (CAD) with low risk of adverse events [2–4]. STEMI was reported in patients undergoing DSE in about 0.1% of cases; most of them had underlying significant coronary arteries [5, 6]. We detail a case of a 39-year-old male who recently had severe hyperthyroidism. He developed chest pain, significant ST elevation, and raised cardiac enzymes following DSE for angina-type chest pain. Coronary angiography showed CAD with mild luminal stenosis.

## 2. Case

**Figure 1:**
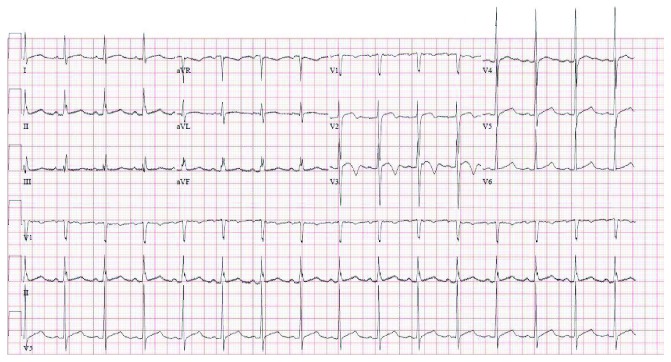


**Figure 2:**
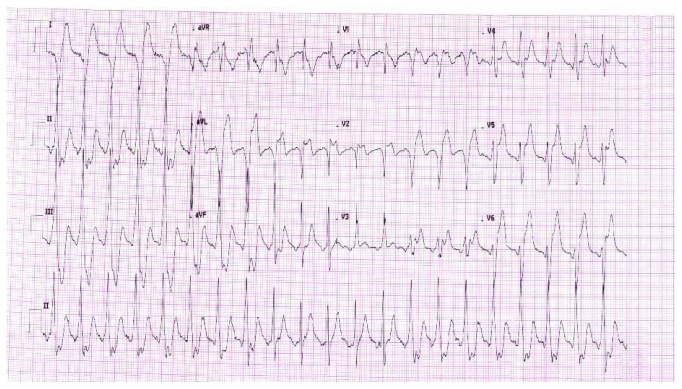


A 39-year-old Asian male, with a recent diagnosis of severe hyperthyroidism and family history of coronary artery disease but no other cardiovascular risk factors, presented with intermittent angina-type chest pain of 8-month duration. He also reported heat intolerance, recurrent palpitation, sweating, watery diarrhea, and weight loss of 10 kg over a 3-month duration. Methimazole and propranolol were initiated two days before the actual presentation. On examination, he had normal heart sounds, no gallop, murmur, or rub tachycardia but with regular and synchronous heart rate, and no clinical signs of heart failure. Neck exam revealed diffuse mild thyroid enlargement and general exam showed fine hand tremors. Electrocardiography on presentation showed biphasic T waves in V1 and V2 (Figure 1). Three sets of cardiac enzymes were negative; TSH was <0.005 mIU/L (0.45-4.5) and T4 was 48.3 pmol/L (9-20) reference and units. Transthoracic echocardiography was normal with good left ventricular function (ejection fraction (EF): 70%) and no regional wall motion abnormalities. As the patient did not tolerate exercise treadmill stress test, he underwent a dobutamine stress echo. Dobutamine was infused at 3-minute intervals, starting with 10 *μ*g/kg and increasing to 20 *μ*g/kg, 30 *μ*g/kg, and 40 *μ*g/kg in addition to 0.5 mg atropine IV until a target heart rate of 153 bpm accounting for 95% of his maximal predicted heart rate. The patient developed severe chest pain and systemic hypotension with a blood pressure of 80/50 mmHg. ST elevation in the anterolateral leads, new RBBB, and short runs of nonsustained ventricular tachycardia (VT) on continuous ECG monitoring were noted (Figure 2).

**Figure 3:**
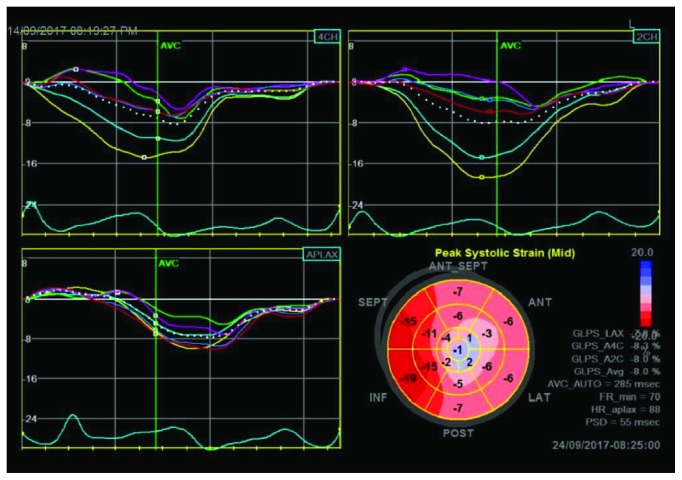


Echocardiography showed new regional wall motion abnormalities in the form of akinesia of the apical, mid anteroseptal, and mid anterior walls and hypokinesia of the mid anterolateral and mid posterolateral walls with a drop of EF to 30%. The patient was transferred to the intensive care unit, and resuscitation with IV fluids led to improvement in his blood pressure and propranolol was uptitrated till heart rate became controlled. Chest pain and ST elevation normalized spontaneously resolved. Cardiac troponin T reached a peak of 1900 ng/L but serial troponins showed progressive improvement. Global longitudinal strain measures (Figure 3) were abnormal in the left anterior descending (LAD) territory (Figure 4). Dual antiplatelets, heparin infusion along with propranolol and statin, started and kept on IV hydrocortisone 100 mg three times daily for 3 days to decrease the possibility of thyroid storm with coronary angiography (CAG). CAG was done 3 days later and showed 40% LAD stenosis proximal to D1 with no evidence of thrombus, myocardial bridging, or coronary vasospasm. Left circumflex artery (LCX) and right coronary artery (RCA) were normal. No provocation test was done and the patient was discharged home in a stable condition on full medications.

The patient showed dramatic improvement of symptoms over the next few days with improvement of the hyperthyroid state and was discharged successfully with minimal symptoms.

## 3. Discussion

Cardiovascular symptoms are the most prominent in patients with hyperthyroidism. The features of angina-type chest pain may be present in about 20% of these patients [7]. The possible mechanism includes underlying atheroma, metabolic effect of thyroxin on the myocardium, secondary arrhythmias [7], and transient coronary artery spasm [8, 9]. Similarly, coronary artery vasospasm with DSE has been reported in about 0.14%-0.4% [5, 10]. Studies [11] have shown that stress echocardiography in hyperthyroidism might lead to the hyperdynamic cardiac state, including worse exercise capacity and impaired vasodilatory and contractile reserves. However, the association between hyperthyroidism and myocardial infarction during DSE has never been reported.

In our patient, there was no history suggestive of coronary vasospasm, and coronary angiogram did not show any significant coronary artery disease. The possible mechanism in our case could be an exaggerated response to dobutamine, in the presence of severe hyperthyroidism and moderate coronary artery lesion.

Similarly, studies [10, 12, 13] have shown STEMI due to oxygen supply-demand mismatch, vasospasm, coronary artery dissection, or coronary artery plaque rupture with subsequent thrombus formation.

Β-blockade may leave *α*-adrenergic vasoconstriction unopposed. Whether administration of propranolol enhances coronary spasm is not well established. Alvarez et al. [14] reported coronary vasospasm after administration of propranolol; however, other studies did not support this theory [15]. Other studies found no advantage of propranolol over diltiazem in preventing coronary spasms [16]. Keeping in mind those previous studies done on euthyroid patients, it may be not applicable to our patient which showed dramatic improvement of symptoms after increasing the propranolol doses.

In our case, we did not see any coronary artery dissection or plaque rupture. It is difficult to rule out a plaque rupture with superimposed thrombus formation, but the transient nature of ST elevation will go against this possibility. Similarly, the coronary angiogram procedure was delayed in order to control his hyperthyroid state firstly, and therefore, the thrombus if present might have been resolved during this time.

## 4. Conclusion

We believe our patient had transient ST-elevation myocardial infarction, secondary to DSE in a poorly controlled hyperthyroid state and moderated coronary artery disease. We would suggest treating hyperthyroidism prior to requesting noninvasive stress test and avoiding invasive coronary angiogram due to thyroid storm risk, based on the clinical picture. Our case also suggests that propranolol may be beneficial in controlling cardiac symptoms and possibly vasospasms in patients with hyperthyroidism.

## Figures and Tables

**Figure 4:**